# Editor's Letter-Box

**Published:** 1902-09-27

**Authors:** 


The House Governor, General Hospital, Birmingham, writes:—"I beg to inform you that we have an X-Ray and Lupus Light Department under the charge of Dr. J. Hall Edwards."

